# Supplementary material for: Complexome profiling of the Chlamydomonas psb28 mutant reveals TEF5 as an early PSII assembly factor
Source: Plant Cell. 2025 Mar 17;37(6):koaf055. doi: 10.1093/plcell/koaf055 (PMC12164590; doi:10.1093/plcell/koaf055)
Supplement: koaf055_Supplementary_Data [file koaf055_supplementary_data.zip › psb28_interactive.zip!/plots/intensity/qualityPlots/peptides/_Cre11.g478800.t1.2.html]

### Cre11.g478800.t1.2

Trivial name:   
SeqCoverage [median]: 17.80  
Mass: 74.61 kDa  
  
 

| Sequence | MissedCleavage | Mass [Da] | wtIntensity | muIntensity | Score |
| --- | --- | --- | --- | --- | --- |
| AAPAPAKEEEK | 1 | 1139.58 | 5232.86 | 1946.74 | 79.82 |
| AAPSTSAPAKEEER | 1 | 1442.70 | 998.32 | 575.14 | 78.49 |
| AEAKAVEVPAGLK | 1 | 1281.73 | 0.00 | 14530.31 | 44.30 |
| AVEVPAGLKPFK | 1 | 1254.73 | 0.00 | 787.39 | 39.92 |
| GASSGNKDIINNLK | 1 | 1429.75 | 0.00 | 315.60 | 33.69 |
|  |  |  | 6231.2 | 18155.2 |  |
